# Ectopic Kidney, a Challenging First Trimester Diagnosis—Case Report and Literature Review

**DOI:** 10.3390/life14111466

**Published:** 2024-11-12

**Authors:** Mircea-Octavian Poenaru, Fernanda-Ecaterina Augustin, Ionuț-Didel Vâlcea, Romina-Marina Sima, Mihaela Amza, Oana Denisa Bălălău, Liana Pleș

**Affiliations:** 1 Department of Obstetrics and Ginecology, University of Medicine and Pharmacy “Carol Davila”, 020021 Bucharest, Romania; mircea.poenaru@umfcd.ro (M.-O.P.); romina.sima@umfcd.ro (R.-M.S.); mihaela.amza@umfcd.ro (M.A.); oana.balalau@umfcd.ro (O.D.B.); liana.ples@umfcd.ro (L.P.); 2 Bucur Maternity, St. John Hospital, 040292 Bucharest, Romania; ionutvalcea@gmail.com

**Keywords:** crossed fused renal ectopia, first trimester morphology, maternal–fetal medicine

## Abstract

(1) Background: Crossed fused renal ectopia is a rare migration and fusion renal anomaly, more frequently affecting males, with an incidence of between 1:2000 and 1:7500 observed at autopsy. (2) Case presentation: This paper presents the case of a 34-year-old woman, IIIG IIIP, who presented to our clinic for a first-trimester screening evaluation. The risk calculation was performed using the Fetal Medicine Foundation’s first-trimester screening software, version 2.8.1. The screening indicated a low risk for aneuploidies, but the ultrasound scan revealed an empty right renal fossa in the standard coronal section. A more detailed axial examination raised the suspicion of crossed fused renal ectopia. No other anomalies were detected. The morphological scans conducted in the second and third trimesters confirmed and upheld the diagnosis initially established in the first trimester. The fetus did not develop any potential antepartum complications. The patient gave birth via caesarean section at 36 weeks to a live female infant, weighing 3000 g, with an APGAR score of 9 at 1 min. Postnatally, the first trimester diagnosis of the renal malformation was confirmed. (3) Conclusions: Crossed fused renal ectopia, probably the rarest renal migration anomaly, can be diagnosed as early as the first trimester of pregnancy.

## 1. Introduction

The renal system contains three successive primitive systems during embryogenesis. The first, the pronephros, is rudimentary and non-functional, located in the cervical region and develops in the fourth week. The second, the mesonephros, evolves from the intermediate mesoderm in the upper thoracic and upper lumbar regions. It appears concurrently with the regression of the pronephros in the fourth week and is the origin of a urogenital ridge by the sixth week. The last structure, the metanephros, develops from the metanephric mesoderm and represents the future renal parenchyma, appearing in the fifth week [1].

The kidney, which starts out in the pelvic region, eventually shifts to a higher position in the abdomen. This upward movement is caused by the straightening of the body’s curvature and the development of the lumbar and sacral regions. In the pelvic region, the metanephros receives its blood supply from a branch of the aorta. As it ascends, it is supplied by arteries originating from progressively higher levels of the aorta. Although some may persist, the lower vessels typically degenerate [1].

Occasionally, a kidney can cross to the opposite side, leading to a condition known as crossed renal ectopia, which may occur with or without fusion [2]. Kidney development relies on the interaction between the ureteric bud and the metanephric blastema. The ureteric bud originates from the lower part of the Wolffian duct, while the metanephric blastema derives from the mesodermal tissue. These move toward each other and interact to develop the kidney and urinary tract.

Caused by the excessive bending and rotation of the embryo’s caudal end, one theory suggests that the ureteric bud may fail to merge with the ipsilateral metanephric blastema. As a result, it shifts toward the contralateral side that is closer. This misdirection leads to both the migrated and the normally positioned ureteric bud, inducing the metanephric blastema to form two kidneys on one side [3,4,5]. Another theory explains that an unusually located umbilical artery may obstruct the normal cephalic migration of the kidney [6].

Regardless of the specific embryological pathway, the result is a deviation in the normal migration of the kidneys from the pelvis to the lumbar fossa, which occurs between the fifth and ninth weeks of gestation. Consequently, this results in renal ectopia without ureteral ectopia, where two kidneys with separate collecting systems are located on one side of the abdomen as one ureter crosses the midline to reach the bladder on the contralateral side.

Crossed renal ectopia (CRE) can be classified into CRE with fusion, where the ectopic kidney crosses to the opposite side and its upper pole is fused to the lower pole of the normally positioned kidney, and CRE without fusion, where the ectopic kidney crosses over in the same manner but without fusion of the kidney poles [2].

Inferior crossed fusion is the most common type of fusion observed in CRE cases. Other less frequent types of CRE with fusion include the following: sigmoid or S-shaped kidney, where the ectopic kidney is positioned below with its pelvis facing outward, while the normally located kidney’s pelvis points inward; lump kidney, where complete fusion creates a mass on one side; L-shaped kidney, where the ectopic kidney is positioned transversely below the normal kidney; disc kidney, where fusion occurs along the inner edges of each pole; and superior crossed fused kidney [7].

CRE occurs in approximately 7.5 out of every 10,000 live births. The condition has a higher incidence in males compared to females, with a male-to-female ratio of 3:2. Left-to-right ectopia is more prevalent in CRE cases, appearing with a frequency of about 2:1 to 3:1 [7].

Crossed fused renal ectopia is a rare condition, with no cases reported as being diagnosed in the first trimester. Due to its similar prognosis to that of the horseshoe kidney, the two conditions are often considered together. This condition is usually identified during a second- or third-trimester scan. Diagnosing crossed fused renal ectopia is important for case management, as it may be associated with well-known complications such as hydronephrosis or other malformations [8].

## 2. Case Presentation

We present the case of a 34-year-old pregnant woman, IIIG IIIP, scheduled in our clinic for a first-trimester ultrasound scan and a biochemical screening at 13 weeks plus 2 days of gestation. The patient had no significant personal or familial medical history. For risk calculation, we used the Fetal Medicine Foundation software dedicated to first-trimester screening, version 2.8.1.

The Fetal Medicine Foundation’s first-trimester screening software, version 2.8.1, organizes data into the following categories: patient information, medical history, ultrasound measurements, biochemistry, and mean arterial pressure (MAP). Patient information includes basic details such as the patient’s name and date of birth. Medical history covers the relevant factors including racial origin, parity, prior deliveries between 16 and 30 weeks, deliveries at or after 37 weeks, current maternal weight and height, smoking status in this pregnancy, presence of diabetes mellitus, chronic hypertension, systemic lupus erythematosus (SLE), antiphospholipid syndrome (APS), preeclampsia in previous pregnancies, the previous delivery of a small baby, a family history of preeclampsia (e.g., patient’s mother), and the method of conception. Ultrasound measurements include required parameters such as examination date, operator, fetal heart activity, crown–rump length (CRL), and nuchal translucency (NT). Additional measurements that are recommended in the first trimester scan include biparietal diameter (BPD), ductus venosus pulsatility index (DV PI), nasal bone, tricuspid Doppler, left and right uterine artery pulsatility index, endocervical length, and detailed fetal anatomy (including skull, brain, heart, stomach, hands, spine, abdominal wall, bladder/kidneys, feet, placenta, amniotic fluid, and umbilical cord). Biochemistry consists of parameters like sample collection date, equipment used, free beta-human chorionic gonadotropin (free β-hCG) in IU/L, pregnancy-associated plasma protein-A (PAPP-A) in IU/L, and placental growth factor (PlGF) in pg/L. Mean arterial pressure (MAP) records the systolic and diastolic blood pressure measured twice in both arms. For accurate blood pressure measurements, an automated and regularly calibrated device should be used. The patient should be seated with arms supported at heart level, and an appropriately sized cuff—small (<22 cm), normal (22–32 cm), or large (33–42 cm) depending on mid-arm circumference—should be selected. After a five-minute rest, blood pressure should be taken in both arms simultaneously, with two readings recorded at one-minute intervals [9].

**Figure 1:**
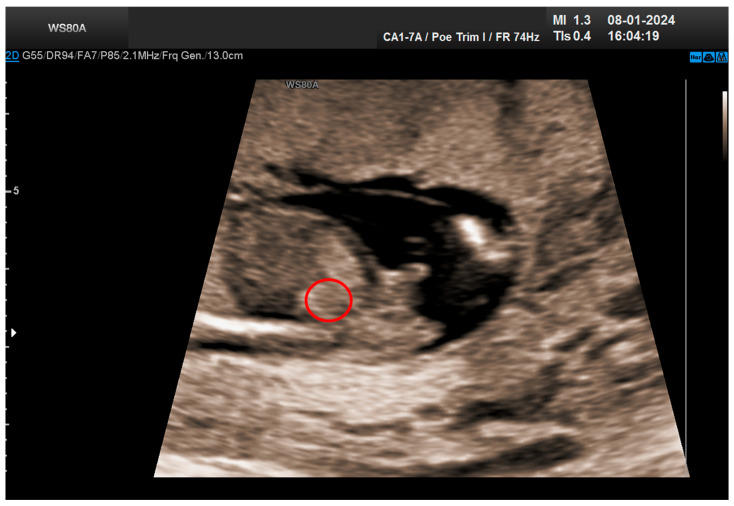
Empty right renal fossa (circled in red)—first-trimester morphology scan at 13 weeks and 2 days.

**Figure 2:**
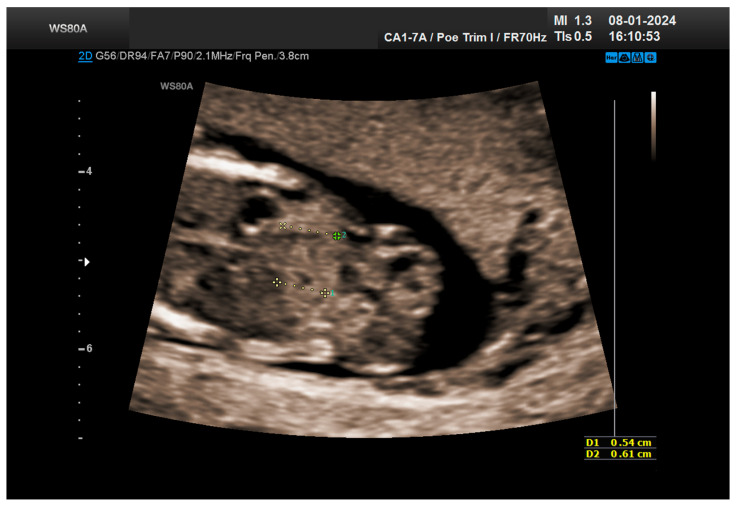
Crossed fused kidneys at 13 weeks.

**Figure 3:**
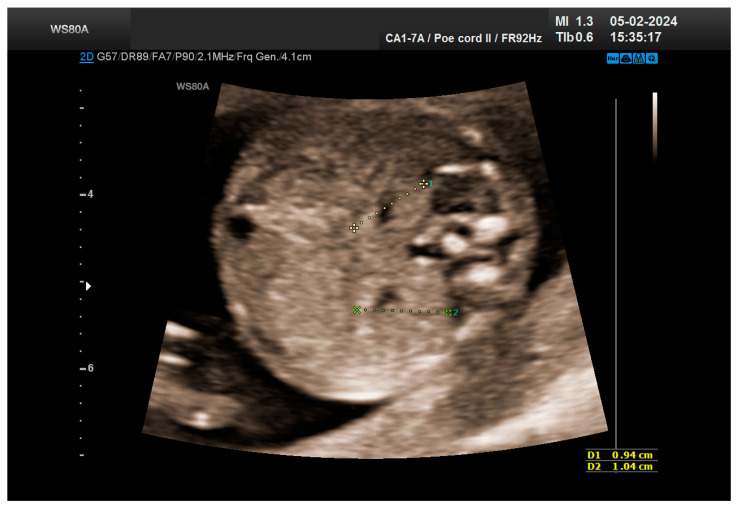
The two kidneys are clearly visualized: the right one, ectopic, fused with the left kidney, normally positioned—ultrasonography revaluation at 17 weeks.

**Figure 4:**
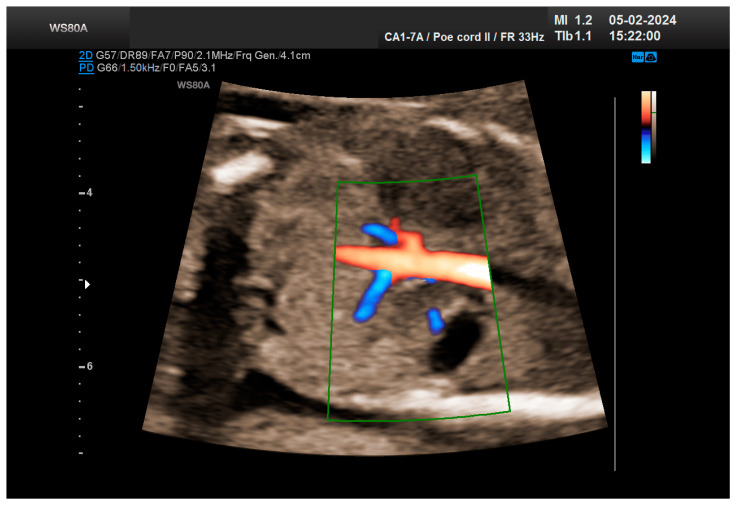
The vascularization of the “renal mass” seems to originate in the aorta. Green box—color Doppler window.

The combined test result indicated a low risk for trisomies, preeclampsia, and intrauterine growth restriction. However, during the ultrasound examination, the absence of the right renal tissue in the right parasagittal sections was noted (Figure 1). Examination of the embryo in axial sections demonstrated the presence of a relatively well-defined acoustically heterogeneous echogenic mass situated anterior to the spine, in sonographic contact with the renal tissue image correctly occupying the left lumbar fossa (Figure 2). Those aspects suggested the existence of a renal anomaly of crossed fused renal ectopia. No other embryonic anomalies were detected, the bladder image was present, and the amniotic fluid volume was normal. The calculated gestational age corresponded to the ultrasound estimate. The suspicion of crossed fused renal ectopia was maintained during a subsequent reevaluation at 17 weeks of pregnancy (Figure 3 and Figure 4).

**Figure 5:**
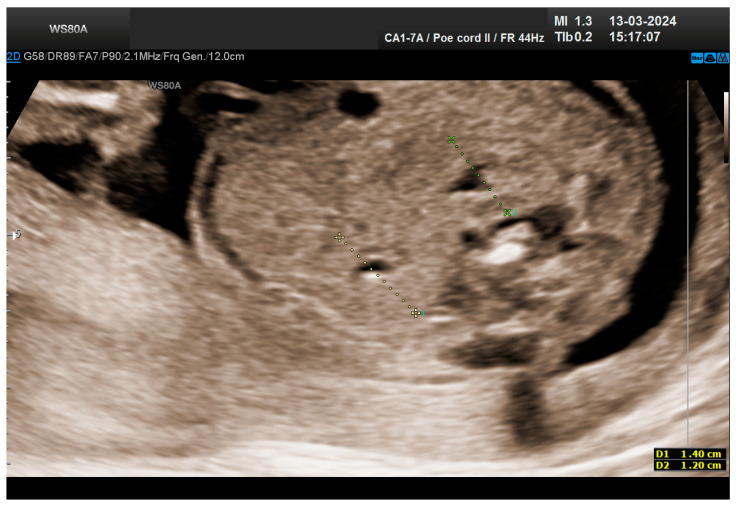
Crossed fused renal ectopia at 22 weeks—second-trimester morphology scan.

**Figure 6:**
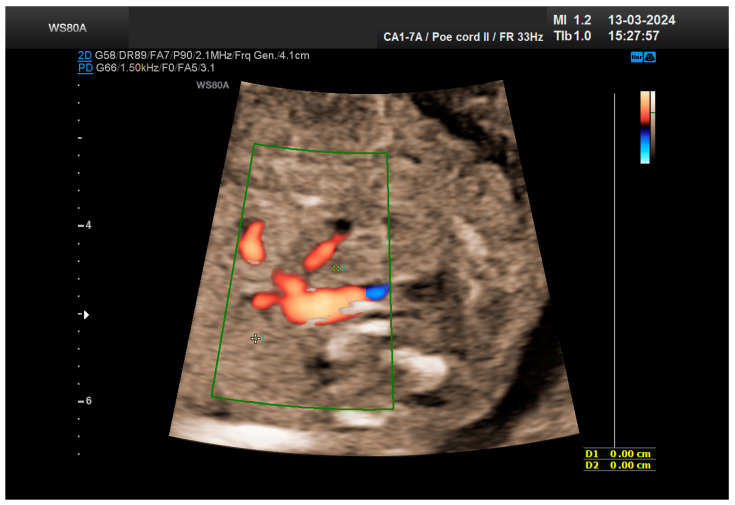
The two kidneys fused with aortic arterial vascularization. Green box—color Doppler window.

The patient returned at a gestational age of 22 weeks for a second-trimester ultrasound screening. During this examination, the suspicion of crossed fused renal ectopia was confirmed. Axial sections revealed a normal kidney image in the left lumbar fossa and another one, anterior to the spine, without reaching the right renal fossa, fused with the normal left kidney (Figure 5). Two sources of arterial vascularization could be identified, both of them with origins in aorta as follows: one correctly originating from the lumbar aorta, serving the normally positioned left kidney, and another appearing to originate on the anterior border of aorta, at the same level, supplying the ectopic right renal tissue mass (Figure 4 and Figure 6). The bladder was present, with normal appearance and volume. No left ureterohydronephrosis was detected. The presence and course of the right ureter could not be identified. The amniotic fluid volume was normal. No other severe or minor anomalies were observed.

**Figure 7:**
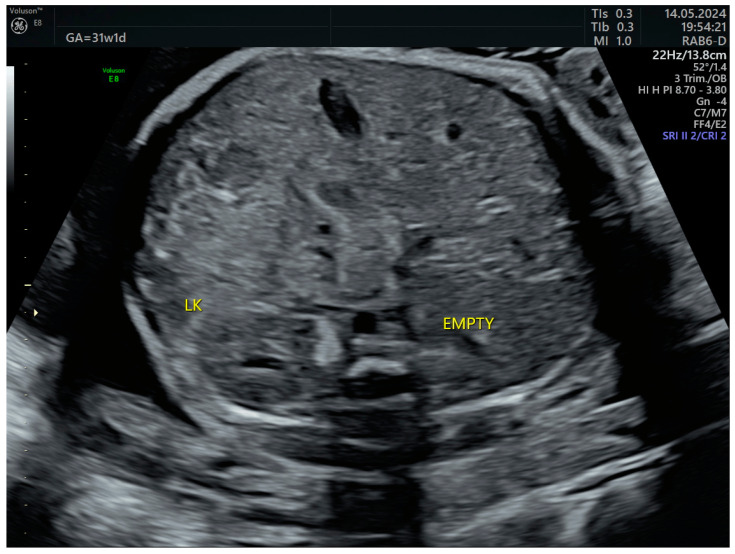
Right lumbar fossa without renal image—third-trimester morphology scan at 31 weeks. LK—left kidney.

**Figure 8:**
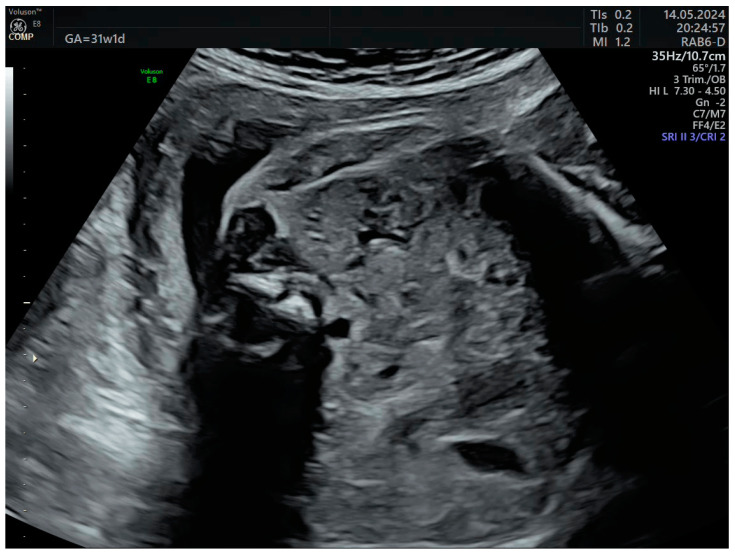
Crossed fused renal ectopia in the third trimester without calyceal or pelvic dilations.

The third-trimester ultrasound evaluation at 31 weeks and 5 days confirmed once again the presence of crossed fused renal ectopia. Although the frequency of the associated changes (hydronephrosis, calyceal dilatations, ureteral dilatations, ureterocele, oligohydramnios, vesicoureteral reflux, etc.) in the third trimester is relatively high, none of these complications were present at the time of examination (Figure 7 and Figure 8). No other anomalies or deviations from the fetal growth curve were recorded, even though, starting from the 28th week, the patient developed diet-controlled gestational diabetes. The final diagnosis was isolated crossed fused renal ectopia.

The patient was monitored clinically and via ultrasound until a gestational age of 36 weeks and 3 days, when she underwent a caesarean section and delivered a live female infant weighing 3000 g, with an APGAR score of 9 at 1 min and APGAR score of 10 at 5 min. The indication for preterm delivery by cesarean section was the imminent risk of uterine rupture on uterus with two scars and uncertain scar quality. The patient had previously delivered at another clinic.

After birth, the newborn was referred for a preliminary urological evaluation, which confirmed the diagnosis that was established in the first trimester. The postnatal follow-up was conducted using an abdominal ultrasound, which confirmed the anomaly. The baby showed no symptoms and is periodically evaluated by the pediatrician through ultrasound scans and urine cultures. Additional investigations will be necessary to thoroughly determine the impact of the anomaly, especially regarding the morphology of the urinary tract and renal excretory function. The parents declined the magnetic resonance imaging (MRI) due to the need for sedation.

## 3. Discussion

Pelvic ectopic kidney has an incidence of between 1/2200 and 1/3000 autopsies [7]. Other data cite an incidence of 1/700 live births [10]. Crossed fused renal ectopia is found at autopsy in between 1/2000 and 1/7500 cases [11]. Crossed fused renal ectopia is more frequently diagnosed in boys, with various studies reporting ratios ranging from 1:2 to 1:5 (female to male). Regarding the direction of the crossed kidney, the left-to-right ectopia is the most commonly encountered variant [12].

In our clinic, during the year 2023, we identified three cases of ectopic kidney, two cases of crossed fused ectopia (1/750), and one case of pelvic ectopic kidney (1/1500). This does not represent the actual incidence of the pathology in the general population since our center is a level III facility, and most cases are referred to us for revaluations and a second opinion. The case presented is the only one identified in the first trimester. The correct standard image that certifies the presence and position of both kidneys from the first trimester is a coronal section that includes the central image of the spine with echogenic areas symmetrically arranged on either side of the spine, sometimes centered by a small hypoechoic area, located caudal to the image of the diaphragm [13]. In cases with superior visibility, Doppler signals of the two renal arteries can also be identified. Clearly, the image is vastly superior when using the transvaginal approach.

Although there are multiple studies on the incidence of ectopic kidney in the pediatric or adult population, very few, if any, have provided data on the incidence of antenatal diagnosis. In a retrospective study from 2019 that followed 100,997 singleton pregnancies presented for first-trimester anomaly scan, the detection rate of non-chromosomal anomalies at all three anomaly scans was analyzed, compared to the diagnosis made postnatally or at the last ultrasound examination in cases of pregnancy termination, miscarriage, or stillbirth. Regarding the ectopic kidney, the detection rate of a pelvic kidney was analyzed. It was diagnosed in five cases, with four in the second trimester and one in the third trimester. No cases were diagnosed in the first trimester [8].

A retrospective study from 2016 investigated the ante- and postnatal prognosis of fetuses diagnosed antepartum with crossed ectopic kidney. The study included 185 fetuses referred between 2005 and 2015 for the revaluation of an empty renal fossa. Crossed ectopic kidney was the diagnosis in 10 (5%) of these cases, with the average gestational age at diagnosis being 24 weeks (range 17–32 weeks). Postnatally, two cases of hydronephrosis that were not visualized antepartum were diagnosed, and another case with vesicoureteral reflux (known to have bilateral hydronephrosis antepartum) was identified [14].

Vesicoureteral reflux (VUR) occurs in 30% of cases with ectopic kidneys and 70% of cases with pelvic kidneys. Moreover, 43% of individuals with renal ectopy also present with additional urologic and genitourinary abnormalities, such as vaginal atresia [15,16]. Another possible complication of an ectopic kidney is hydronephrosis. This can occur secondary to primary ureteropelvic junction obstruction, significant vesicoureteral reflux, or extrarenal pelves and calices with renal malrotation producing apparent ureteropelvic junction obstruction [7].

Crossed fused renal ectopia can lead to complications secondary to obstruction in almost 50% of cases, such as pyelonephritis and nephrolithiasis, as shown in a 2013 case series, which included six patients diagnosed with crossed fused renal ectopia between 1997 and 2010 [17]. A 2019 study that included 36 pediatric cases of crossed fused renal ectopia showed that the most common presentation was urinary tract infection (13 cases, 36%), especially in children under 5 years old. The diagnosis was often made during abdominal ultrasound evaluations in patients with cardiac anomalies, anorectal malformations, or tracheoesophageal fistula (eight cases, 22%). In children over 5 years old, the most frequent presentation was nonspecific abdominal pain. Other common presentations included hematuria and incidental detection. The most frequent associated congenital anomaly was anorectal malformation (nine cases), with four of these children diagnosed with the Vertebral anomalies, Anal atresia, Cardiac malformations, Tracheoesophageal fistula, Renal anomalies, Limb abnormalities (VACTERL) association. Additionally, two children had isolated sacral agenesis, and there were single cases of undescended testes, hypospadias, and achalasia cardia, respectively [12]. Due to the rarity of this anomaly, there are no large-scale studies evaluating the long-term complication rates and potential risk factors for unfavorable prognosis. Further studies on larger patient cohorts are needed in the future.

In our case, the anomaly had no associations and no consequences on the urinary tract, at least during the pregnancy, leading to the diagnosis of isolated crossed fused renal ectopia. In the absence of other associations and considering the literature data, we did not offer a genetic test during pregnancy (in our country, only a conventional karyotype is reimbursed prenatally).

The long-term prognosis of renal ectopia is influenced by the occurrence of complications, with the most frequent being hydronephrosis and the formation of renal calculi. Renovascular hypertension has been observed in a few cases of renal ectopia, but no causal relationship has been established until now. The risk of malignancy is not higher in patients with renal ectopia. Regarding renal function, one study indicated decreased renal function (38%, 33–43% CI 95%) upon radiological examination with dimercapto succinic acid (DMSA). However, this method is limited by the possibility of inaccurate results due to radiation uptake by the pelvis from the radionuclide. Overall renal function was normal, with a mean follow-up duration of 7.7 years [18]. The high rate of vesicoureteral reflux (VUR) and relatively reduced function, combined with a susceptibility to drainage problems and stone formation, are potential risk factors for the loss of renal function over time. Therefore, these children require long-term follow-up and surveillance [19,20,21]. Crossed fused ectopic kidney may be associated with additional abnormalities, including uterine malformations, imperforate anus, and skeletal anomalies. Less commonly, it may also be linked to septal heart defects, hypospadias, cryptorchidism, and urethral valves [22].

Among the malignant tumors associated with renal fusion anomalies, renal cell carcinoma was the most frequent type of neoplasm. However, its incidence does not exceed that of the general population, and the prognosis is the same as for a normally located kidney. Transitional cell carcinoma was more frequently diagnosed in patients with horseshoe kidneys, having a worse prognosis due to late diagnosis [23].

The Vertebral anomalies, Anal atresia, Cardiac malformations, Tracheoesophageal fistula, Renal anomalies, Limb abnormalities (VACTERL) association requires the presence of at least three of the listed congenital anomalies. A multicenter study from 2020, which included 501 cases diagnosed with the association in the Joint Research Centre-European Surveillance of Congenital Anomalies (JRC-EUROCAT) central database (which included patients born between 1980 and 2015), showed a prevalence of renal anomalies in 51% of cases. Among the major VACTERL renal features, the spectrum of ectopic kidney, including horseshoe kidney and fused kidney, was found [24].

## 4. Conclusions

Renal ectopy is relatively common, occurring in approximately between 1 in 700 and 1 in 5000 live births. Prenatal diagnosis is typically achieved through ultrasound screening during the second and third trimesters. However, careful examination in the standard coronal and axial sections can raise suspicion of renal ectopia as early as the first trimester of pregnancy. To the best of our knowledge, this is the first case of a crossed fused renal ectopia diagnosed in the first trimester of pregnancy. An incorrect coronal image at the lumbar spine level, missing one or both renal images, can and should draw attention to a possible renal ectopia or agenesis.

Crossed fused renal ectopia, probably the rarest renal fusion anomaly, can be diagnosed as early as the first trimester of pregnancy. The prenatal diagnosis of renal ectopy is crucial due to the risk of late and recurrent infectious complications, which can ultimately lead to the loss of renal function.

## Data Availability

The data used in this study are available from the corresponding author, and the authors can share the information if there is a reasonable request.
